# *In silico* Gene Set and Pathway Enrichment Analyses Highlight Involvement of Ion Transport in Cholinergic Pathways in Autism: Rationale for Nutritional Intervention

**DOI:** 10.3389/fnins.2021.648410

**Published:** 2021-04-20

**Authors:** Audrey Olson, Fuquan Zhang, Hongbao Cao, Ancha Baranova, Margaret Slavin

**Affiliations:** ^1^Department of Nutrition and Food Studies, College of Health and Human Services, George Mason University, Fairfax, VA, United States; ^2^School of Systems Biology, College of Science, George Mason University, Manassas, VA, United States; ^3^Department of Psychiatry, Nanjing Medical University, Nanjing, China; ^4^Department of Psychiatry, Shanxi Medical University, Taiyuan, China; ^5^Research Centre for Medical Genetics, Moscow, Russia

**Keywords:** acetylcholine, dietary choline, autism, sensory processing, cholinergic, gene set enrichment analysis

## Abstract

Food is the primary human source of choline, an essential precursor to the neurotransmitter acetylcholine, which has a central role in signaling pathways that govern sensorimotor functions. Most Americans do not consume their recommended amount of dietary choline, and populations with neurodevelopmental conditions like autism spectrum disorder (ASD) may be particularly vulnerable to consequences of choline deficiency. This study aimed to identify a relationship between ASD and cholinergic signaling through gene set enrichment analysis and interrogation of existing database evidence to produce a systems biology model. In gene set enrichment analysis, two gene ontologies were identified as overlapping for autism-related and for cholinergic pathways-related functions, both involving ion transport regulation. Subsequent modeling of ion transport intensive cholinergic signaling pathways highlighted the importance of two genes with autism-associated variants: *GABBR1*, which codes for the gamma aminobutyric acid receptor (GABA_B__1_), and *KCNN2*, which codes for calcium-activated, potassium ion transporting SK2 channels responsible for membrane repolarization after cholinergic binding/signal transmission events. Cholinergic signal transmission pathways related to these proteins were examined in the Pathway Studio environment. The ion transport ontological associations indicated feasibility of a dietary choline support as a low-risk therapeutic intervention capable of modulating cholinergic sensory signaling in autism. Further research at the intersection of dietary status and sensory function in autism is warranted.

## Introduction

Sensory processing dysfunction, which is commonly experienced by persons diagnosed with autism spectrum disorder, extends across auditory, visual, pressure, temperature, pain, vestibulary, and interoceptive processing domains (Craig, 2002; Ashburner et al., 2008; Case-Smith et al., 2015; Boterberg and Warreyn, 2016; Crasta et al., 2016). Some features of sensory processing, including heightened sensitivity to sound (hyperacusis), appear to be prevalent in the majority of the autistic population (Wilson et al., 2017; Williams et al., 2020). All types of sensory processing rely on sensory gating—the ligand-activated, ion-channel mediated pathways built upon cholinergic neurotransmission signals. Acetylcholine, a primary neurotransmitter ligand in these pathways, plays a key role in cognitive function, memory, learning, and sensory processing signal transduction. Acetylcholine is made in an enzymatic reaction between acetyl-CoA and choline, which is facilitated by the choline acetyltransferase (Oda, 1999). Neuronal molecules associated with acetylcholinergic signal transmission, including the nicotinic receptor family, are heavily implicated in propagation of auditory signals through respective transmission pathways (Simmons et al., 2011; Bertrand et al., 2015; Deutsch et al., 2015; Dineley et al., 2015; Sinkus et al., 2015; Gillentine et al., 2017). Research of cholinergic function in autism has led to clinical trials examining efficacy of cholinergic enhancement and/or acetylcholinesterase inhibitors—more commonly used in Alzheimer’s disease—to address social and cognitive aspects of autistic behaviors (Bentley et al., 2003; Hertzman, 2003; Nicolson et al., 2006; Ghaleiha et al., 2014; Karvat and Kimchi, 2014; Rossignol and Frye, 2014; Eissa et al., 2018). However, cholinergic support to sensory processing in autism remains less examined. Recent developments in autism genome-wide association studies (GWAS) have provided estimates of an overlap of autism-related gene sets with those involved in sensory processing and in cholinergic signal transmission.

While humans can make small amounts of choline endogenously, the replenishment of the pool of acetylcholine available in the human body depends heavily upon dietary choline intake (Gibellini and Smith, 2010). In the United States, nearly nine in 10 Americans over 2 years of age do not meet recommended daily consumption levels of dietary choline. Moreover, an estimated 60–93% of children on the autism spectrum are not meeting their adequate intake (AI) level for choline either, even as experts in choline metabolism continue to emphasize its criticality for nutrition across the lifespan, particularly at critical stages of neurodevelopment (Caudill, 2010; Hamlin et al., 2013; Wallace and Fulgoni, 2016; Blusztajn et al., 2017; Ganz et al., 2017; Wallace et al., 2018, 2019; Zeisel, 2019). In rodents, choline-deficient diets are associated with decreased levels of brain acetylcholine (Cohen and Wurtman, 1976). In both rodents and humans, choline deficiency is associated with impaired sensory gating function (Wu et al., 1993; Fendt and Koch, 1999; Stevens et al., 2008; Knott et al., 2014; Swerdlow et al., 2016). Given possible risk of choline underconsumption and/or its deficiency in humans, and the known impact of choline deficiency upon brain acetylcholine and sensory function in rodents, further investigations into the potential impact of choline underconsumption upon sensory processing are warranted, particularly with respect to those on the autism spectrum. Such research may eventually open an avenue for modulation of sensory processing through nutritional interventions.

To explore the potential relationship between autism spectrum disorders (ASDs) and choline intake, we conducted an enrichment analysis of gene sets associated with ASD and cholinergic pathways, and constructed the model reflecting this relationship. Results of our study highlight the potential impact of dietary choline deficiency upon cholinergic signaling within the genetic context of autism.

## Materials and Methods

### Overview of Workflow

This study has focused on gene ontologies cataloged in the Gene Ontology (GO) database for enrichment analysis of an overlap between two gene sets, one for cholinergic pathways and one for autism-associated genes. Gene ontological enrichment analysis was conducted within the Pathway Studio environment (Elsevier, Inc.)^1^ between December 2019 and March 2020. The Pathway Studio database contains functional relationships and pathways of mammalian proteins, including human, mouse, and rat genes. It contains over 1.4 million entities of 14 well-defined categories, including cells, proteins, disease, and small molecules, and more than 13.4 million relationships among these entities. The database includes over 24 million PubMed abstracts and 3.5 million Elsevier and third party full-text papers. Using the natural language processing (NLP) functionality of Pathway Studio, intersections of these gene sets’ pathways were rendered graphically, with connections indicating directional relationships being supported by the current scientific literature. This exploratory analysis and figure development which employed in part some of Pathway Studio’s graphical capabilities took place between March 2020 and November 2020. Since many of the autism-associated genes in the GWAS study have been recently identified as autism-associated only within the past 2 years, this gene/pathway modeling effort was supported by secondary analyses, including use of predictive tools in splice site identification.

### Identification of Ontologies Associated With Each Gene Set, and Shared Ontologies

To identify GO database pathways shared between cholinergic pathways and autism-associated genes, gene set enrichment analysis was conducted. Molecular Signatures Database v7.1 (MSigDB)—one of the most widely used repositories of thoroughly annotated gene sets, used in research on both neurological conditions and on cholinergic pathways, specifically—was chosen as a precurated resource for gene sets connected with cholinergic activity (Tan et al., 2010; Turcan et al., 2010; Liberzon et al., 2011; Koker et al., 2018; Schijven et al., 2018; Zhang et al., 2019). A key word search for “cholin^∗^,” identifying any word beginning with cholin- (choline, cholinergic, cholinesterase, etc.), was used to identify cholinergic-relevant gene sets encompassing a total of 345 genes, which constituted the cholinergic activity gene set for this ontological enrichment analysis. An autism gene set containing 47 genes from a recent cross-trait genome-wide meta-analysis (Wu et al., 2020) was used because it identified more risk genes for ASD compared with another GWAS study for ASD, thus providing enhanced statistical power for the present study (Grove et al., 2019).

Gene ontology association analysis was conducted with each respective set of genes within Pathway Studio. The lists of ontologies assigned to each of these gene sets were trimmed at approximately the lowest 100 *p*-values (*p* < 0.01). A shift in the magnitude of *p*-values, nearest the lowest 100 *p*-values, served as the final determinant of cut-off for each respective gene sets’ ontology lists. The overlaps were identified using the Venn diagram web tool, Venny 2.1 (Oliveros, 2007–2015).

Genes from the autism gene set were then examined for their potential upstream or downstream relationships with acetylcholine, with additional attention paid to potential roles related to the identified shared ontologies, as they were related to sensory processing and cholinergic sensory signal transmission. The identification of upstream and downstream elements and related pathways was conducted using Pathway Studio’s natural language processing tools within its menu architecture, which generate indices of related entities (genes, proteins, physiological conditions, etc.) per tool sorting specifications. The main two tool specifications used in this exploratory analysis were the Pathway Studio options to view first degree connections (known in Pathway Studio as Direct Interactions) and to view the shortest path between entities within the literature (known in Pathway Studio as Shortest Path). Both of these tool specifications were the primary means of building a broader understanding and figure illustration of literature-supported cholinergic networks related to both genes within the autism gene set and to theoretical cholinergic signal disruption related to dietary choline deficiency.

### Gene Set Pathway Intersection Analysis of Shared Ontologies

Shared ontologies and associated pathways were rendered graphically using Pathway Studio’s NLP-driven platform. Additionally, certain elements of interest from the ontological overlap and the intersection between pathways involving genes from these sets were inserted.

Graphical models were developed to examine specific pathways or networks with key autism-associated genes, and respective genetic variants which may alter choline and/or acetylcholine levels available for neuronal sensory signal transport. Each instance of a relationship identified by NLP-based word triplet identification was graded by Pathway Studio in terms of confidence levels of 1–3, with a maximum confidence level of 3 being defined as an entity-to-entity relationship supported by at least three peer reviewed publications reflected by the graphical rendering. For this study and related models, the confidence threshold for including elements in graphical representation was set at 3.

### Supplemental Analyses for Model Refinement

Splice site identification algorithm Alternative Splice Site Predictor (ASSP) (Wang and Marín, 2006) was used to examine potential changes in the splicing expression which may arise from intronic variants located within autism-associated genes.

## Results

### Shared Gene Ontologies

**FIGURE 1:**
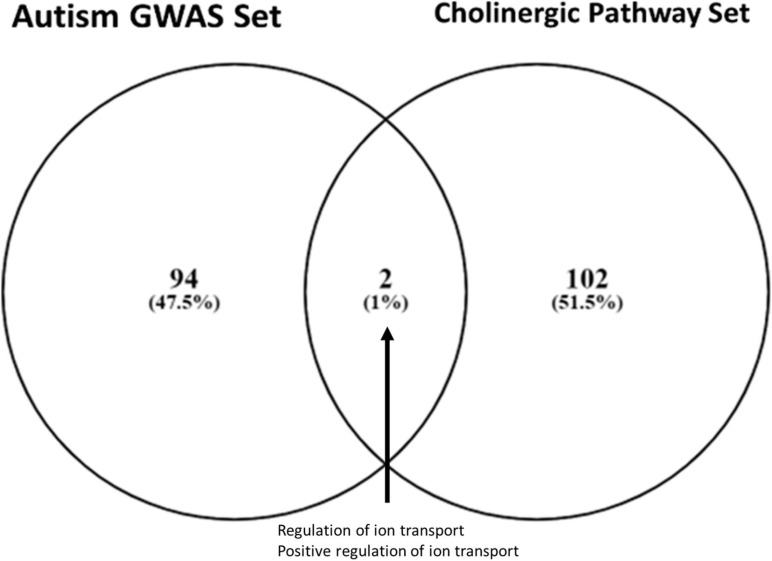
Gene ontology overlap between autism and cholinergic metabolism gene sets. Overlap between an autism-associated genome-wide associated studies gene set (Wu et al., 2020) and cholinergic pathways gene set (MSigDB v7.1) was identified using Venny 2.1. The two shared ontologies were (1) regulation of ion transport and (2) positive regulation of ion transport, identifying ion transport functionality as a critical area of enrichment for further analysis.

Two ontologies were shared between the cholinergic pathway and the autism GWAS gene sets (*p* < 0.05), namely, those for ion transport regulation and positive ion transport regulation (Figure 1). These ion-transport-related pathways are associated with sensory processing functionality across broad spectrums of the relevant domains, including nociception, tactile response, vestibular reflex, startle response, pupillary light reflex, and sensory gating in general.

Within these two ion transport regulation ontologies, two common genes were identified as implicated in neuronal signal transmission: the gene *GABBR1*, which encodes the gamma aminobutyric acid membrane receptor GABA_B__1_, also known as “GABA receptor” elsewhere in the literature, and the gene *KCNN2*, which encodes potassium intermediate/small conductance calcium-activated channels of SK2 type. The latter channels are often co-located with cholinergic receptors and are capable of modulating cholinergic signaling through membrane repolarization.

### Analysis of the Intersection of Autism-Related Cholinergic Gene Sets

**FIGURE 2:**
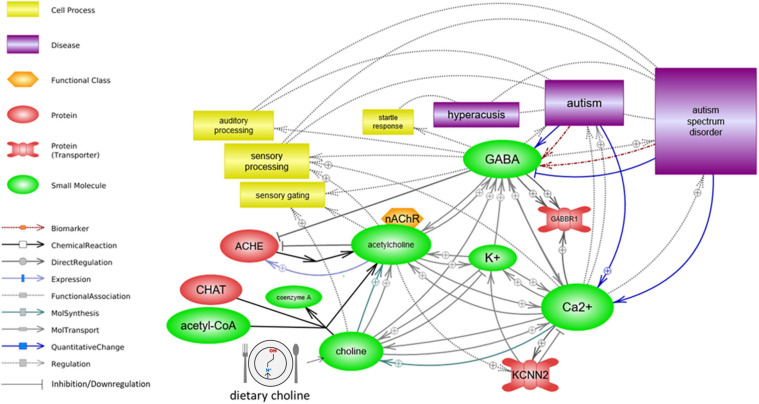
Sensory processing is associated with autism functionally, in the context of ion transport and cholinergic signaling. Acetylcholine regulates both the ion transport and the GABA signaling. A deficiency in its precursor, dietary choline, may impact ion transport, GABA⇒*GABBR1* signaling, and *KCNN2*/SK2 channel function, either or all of which may result in altered sensory processing function.

Analysis with Pathway Studio’s graphical pathway rendering platform identified regulatory and functional relationships between acetylcholine, ion transport/ion levels, *GABBR1* and *KCNN2* activity, and aspects of sensory processing. All depicted relationships are supported by experimental evidence extracted from the scientific literature (Figure 2).

**FIGURE 3:**
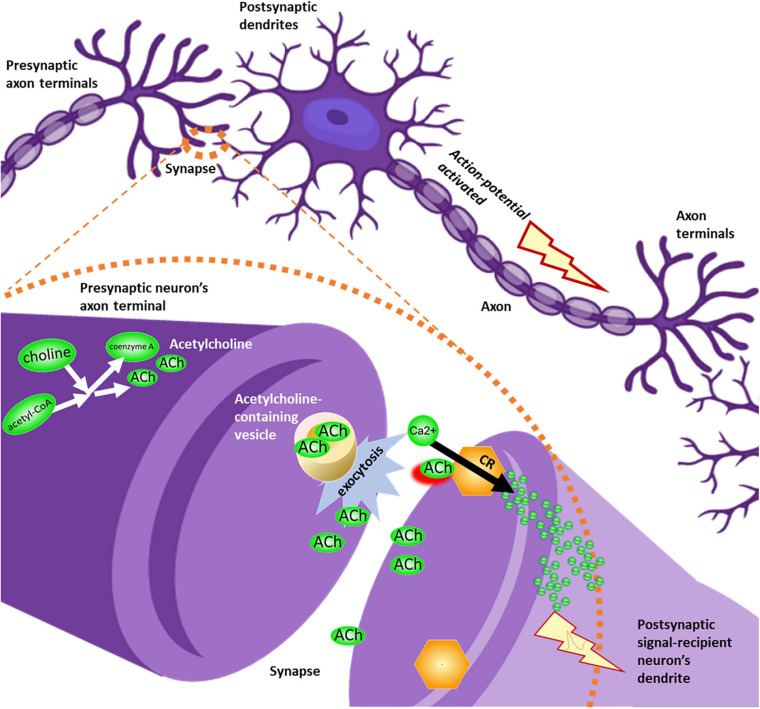
Overview of acetylcholinergic signaling. Acetylcholine (ACh), sourced from precursors choline and acetyl-CoA, leaves the presynaptic neuron’s axon terminal through vesicle-mediated exocytosis. Acetylcholine molecules diffuse through the synapse, until binding with cholinergic receptors (CR) in the membrane of the postsynaptic signal-recipient neuron’s dendrite. This binding event permits influx of calcium ions into the dendrite. Ionic membrane depolarization activates an action potential, causing an electrical signal to be propagated down the length of the axon in a series of ion transport events, until the signal reaches the axon terminal and is translated again into neurotransmitter signals destined to reach the next neuron’s dendrite.

Choline and acetylcholine were linked to ion transport function across several pathways, with the most direct implication being the neuronal influx of membrane depolarizing calcium resulting from the binding of acetylcholine to cholinergic receptors, such as the alpha-7 nicotinic receptor. This transition from chemical signal transduction (release of acetylcholine into the synapse which binds with the nicotinic receptor on the dendrite of the signal recipient neuron) to ionic signal transduction down the axon is a core aspect of sensory signal pathway functionality (Figure 3).

**FIGURE 4:**
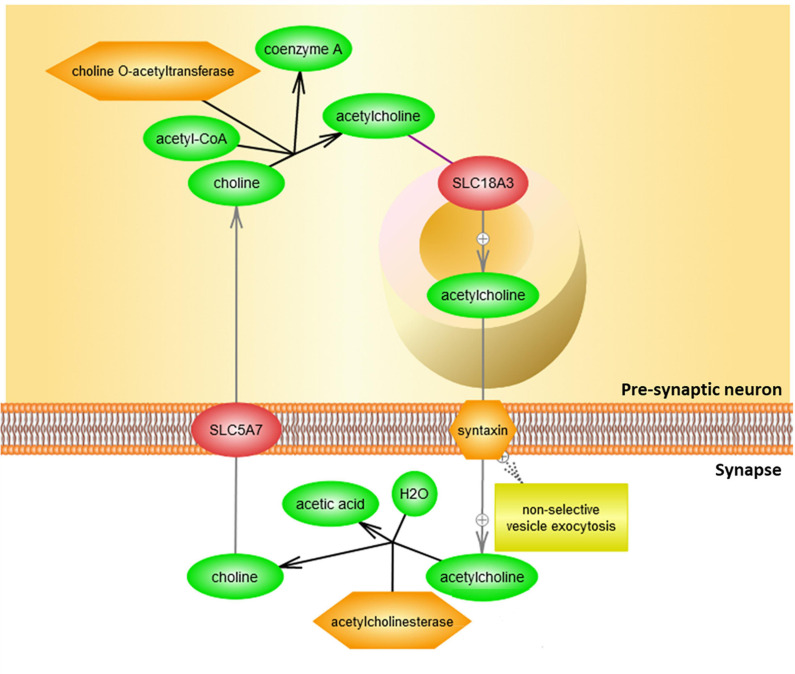
Clearance of acetylcholine from the synapse by acetylcholinesterase. Acetylcholine (ACh) is delivered to the synapse as a result of vesicle exocytosis from pre-synaptic neuronal signaling and a release of the recycled acetylcholine from the postsynaptic neuron’s cholinergic receptors. In the synaptic cleft, acetylcholinesterase (ACHE) then degrades acetylcholine. Resulting choline is then taken back by the presynaptic neuron to be recycled in order to make more acetylcholine for future signaling. This figure was extracted from Pathway Studio’s curated pathways and was edited according to the needs of this study.

Binding of acetylcholinergic receptors is modulated through multiple means, both preemptive and *ex post facto*. The enzyme acetylcholinesterase functions by cleaving acetylcholine in the synapse, including either acetylcholine recently made available for signal transduction, or acetylcholine released from membrane-bound acetylcholinergic receptors in the signal recipient neuron (Figure 4).

**FIGURE 5:**
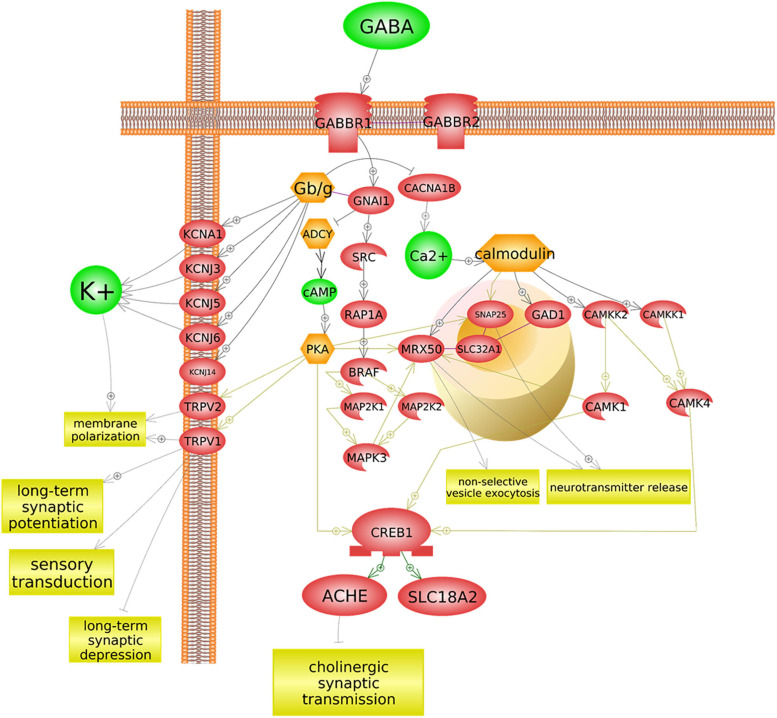
*GABBR1* may influence acetylcholinesterase regulation. *GABBR1* is an autism-associated gene that codes for a membrane-bound protein (GABA_B__1_) (Wu et al., 2020). The binding of the neurotransmitter GABA to GABA_B__1_ has several implications for cholinergic signaling, sensory signal transduction, and ion transport, across multiple cascades. As depicted here, a typical GABA/*GABBR1* binding cascade inhibits adenylyl cyclases (ADCY), thereby preventing positive upregulation of acetylcholinesterase (ACHE). This figure was extracted from Pathway Studio’s curated pathways and was edited according to the needs of this study.

**FIGURE 6:**
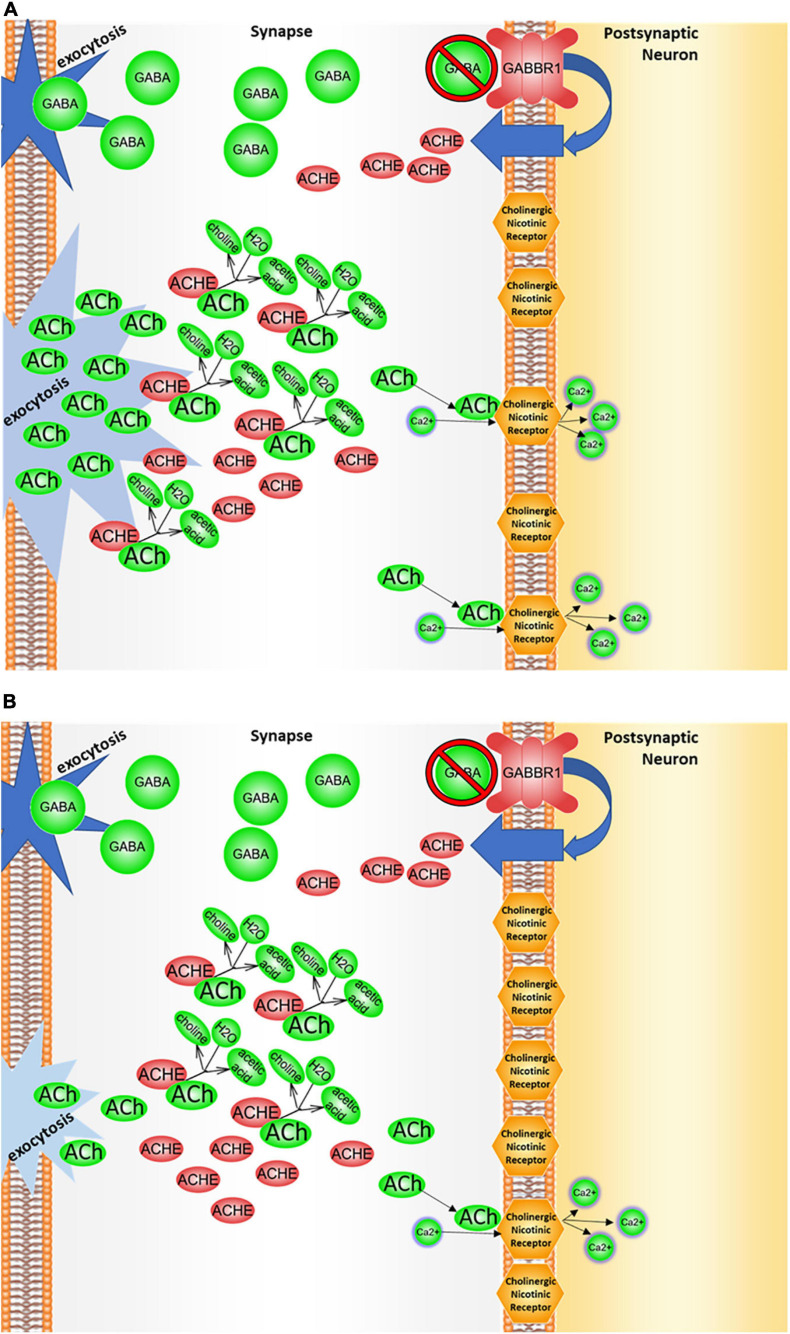
*GABBR1*/ACHE regulation. **(A)** In the presence of *GABBR1* variant. The autism-associated *GABBR1* variant may affect levels of acetylcholinesterase (ACHE) in the synaptic cleft by decreasing either total levels of *GABBR1* expression and/or function of its gene product, the GABA_B__1_ receptor. Under this condition, the cascade that normally downregulates acetylcholinesterase expression may be suppressed. Because of that, larger amounts of the enzyme are produced, and the acetylcholine degrades at an elevated rate, resulting in less acetylcholine in the synaptic cleft and less signaling through the cholinergic receptor. **(B)** In the presence of *GABBR1* variant and a dietary choline deficiency. When choline levels are deficient, less acetylcholine (ACh) is available in the neurons for signaling, and a decrease in cholinergic binding/signaling attributed to *GABBR1* variant would be exacerbated.

The regulatory role of GABA-ergic binding in acetylcholinergic activity is nuanced. Typically, GABA is released from the signal-transmitting neuron synchronously with acetylcholine through separate GABA-specific vesicles, which serve as an immediate signal modulator. The subsequent cascade that arises from GABA binding to the GABA_B__1_ receptor suppresses further upregulation of acetylcholinesterase (ACHE) and preserves acetylcholine in the synaptic cleft from destruction. The signal propagated through the GABA_B__1_ receptor is, therefore, hypothesized to modulate an availability of acetylcholinesterase within the cleft. In other words, GABA-ergic suppression of ACHE upregulation may lead to decrease of synaptic acetylcholine degradation, leaving more acetylcholine available for cholinergic signal transmission. This cascade’s impact upon synaptic acetylcholine levels is depicted in Figure 5. A hypothetical impact of genetically determined differences in GABA–GABA_B__1_ binding activity upon cholinergic sensory signal modulation is depicted in Figure 6A.

**FIGURE 7:**
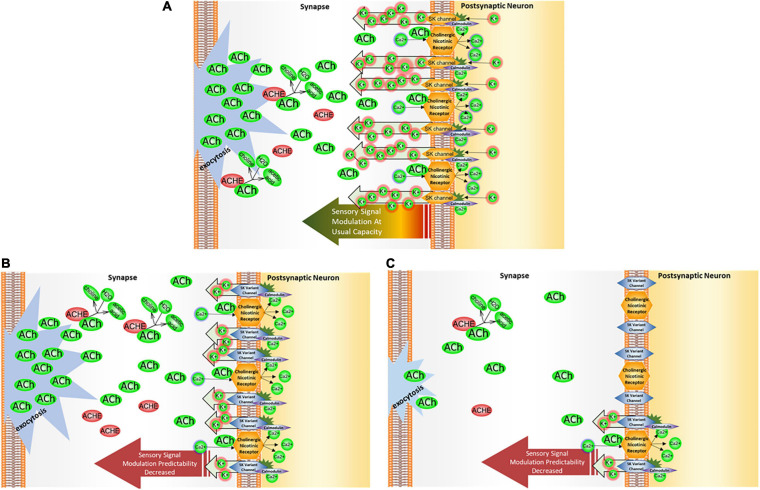
*KCNN2/*SK2 impact on cholinergic signal modulation. **(A)** SK2 channel operation. SK2 channels operate in tandem with a propagation of cholinergic signal as they counter the membrane depolarization brought about by calcium influx through cholinergic receptors. The calcium influx activates the calmodulin domains on SK2 channels, permitting passage of potassium back into the synapse to restore membrane hyperpolarization. Thus, SK2 channels serve a critical role in helping a neuron to recover and re-hyperpolarize before receiving the next cholinergic signal. **(B)** In the presence of *KCNN2*/SK2 variant. When SK2 channel binding activity and/or functionality is altered, the sensory signaling modulation could either be *too efficient*—that is, the membrane repolarizes too soon due to overflow of K + ions back into the synapse, or *not efficient enough*, with membrane taking too long to repolarize, leaving the neuron less ready to process any subsequent signals transmitted from the presynaptic neuron. In either of these two conditions, the neuron’s capacity to modulate sensory signaling through membrane repolarization may be diminished. **(C)** In the presence of *KCNN2*/SK2 variant and a dietary choline deficiency. When choline levels are deficient, less acetylcholine (ACh) is available in the neurons for signaling, and a post-signal repolarization may be less amenable to modulation by SK2 channels. When these channels are working in a suboptimal regimen due to the presence of *KCNN2* variant, modulation leeway may be curtailed even further.

Other modulators of acetylcholinergic signal transmission include SK2 ion channels, which counter the membrane depolarization in the signal recipient neuron (Figure 7A). These types of membrane repolarizing ion channels prepare the signal recipient neuron to receive other action potential-activated sensory signals immediately after processing the preceding signal.

In part, the magnitude of the sensory signal depends upon SK2 channels’ ability to effectively counter membrane depolarization in the wake of a cholinergic signal, thus, serving as an *ex post facto* modulator of cholinergic activity. The autism-associated *KCNN2* variant may either change the level of the baseline SK2 channel expression or lead to the alteration of the splicing, and the shift of the open reading frame. In either case, sensory signal stands to be impacted. These hypothetical impacts of a *KCNN2* genetic variant upon cholinergic sensory signal can be seen in Figure 7B.

The final modeling stage sought to evaluate and depict these hypothesized sensory signaling outcomes in light of a deficiency sufficient to reduce available acetylcholine. The intersection of dietary choline deficiency with *GABBR1-*related pathways can be seen in Figure 6B, and the intersection of dietary choline deficiency with *KCNN2-*related pathways can be seen in Figure 7C.

### Supportive Evidence for KCNN2 Variant as a Modifier of Splicing

To examine a possible change in splicing patterns attributable to autism-associated variants in *GABBR1* and *KCNN2*, both the wildtype loci and the variant loci representing each of these two genes were compared using the Alternative Splice Site Predictor (ASSP) tool. While the (rs740883) SNP-related differences in predicted splicing patterns of *GABBR1* were minimal, the *KCNN2* variant rs13188074 yielded a high confidence prediction of a lowered strength within the splice site between the seventh and eighth exons (Table 1), indicating the possibility of a lower rate of splicing than may occur in the wildtype version of this site.

**TABLE 1:** The Alternative Splice Site Predictor (ASSP) tool (Wang and Marín, 2006) determined short windows containing the loci for each of the *GABBR1* and *KCNN2* autism-associated genes.

					Activations**		
Gene/Variant	Putative splice site	Sequence	Score*	Intron GC*	Alt./Cryptic	Constitutive	Confidence**
*GABBR1* Wildtype	Alternative isoform/cryptic acceptor	CACGCTCCAGCCATGCTG**A**A	4.170	0.614	0.847	0.127	0.827
*GABBR1* Variant	Alternative isoform/cryptic acceptor	CACGCTCCAGCCATGCTG**T**A	4.170	0.614	0.888	0.106	0.880
*KCNN2* Wildtype	Alternative isoform/cryptic donor	TAAGCTTATGGTA**A**AAGAGT	7.508	0.429	0.617	0.294	0.523
*KCNN2* Variant	Alternative isoform/cryptic donor	TAAGCTTATGGTA**G**AAGAGT	5.195	0.443	0.778	0.160	0.795

In the case of the *GABBR1* wildtype/variant comparison, no difference in scoring of splice site strength was calculated, and only a slight shift in confidence occurred of this window containing an alternative isoform/cryptic acceptor. However, the *KCNN2* wildtype/variant comparison indicated a lower score for splice site strength, in its classification as an alternative isoform/cryptic donor, and this lower score attained a higher confidence rating of 0.795, compared to the 0.523 confidence rating that ASSP assigned to the splice site strength score for the window containing *KCNN2* wildtype, also classified as an alternative isoform cryptic donor.

*Scores of the preprocessing models reflecting splice site strength, i.e., a PSSM for putative acceptor sites, and an MDD model for putative donor sites. Intron GC values correspond to 70 nucleotides of the neighboring intron.

**Activations are output values of the backpropagation networks used for classification. High values for one class with low values of the other class imply a good classification. Confidence is a simple measure expressing the differences between output activations. Confidence ranges between zero (undecided) and one (perfect classification).

*Bolded letters in the sequences indicate the single nucleotide polymorphism difference between wildtype and variant.*

## Discussion

This paper presents the results of a gene set enrichment analysis focusing on shared gene ontologies between an autism-associated gene set obtained through meta-analysis of GWAS and a gene set centering on cholinergic function, which has been collated using a combination of curated gene sets in the Molecular Signatures Database (MSigDB) (Liberzon et al., 2011; Liu et al., 2020). Our study identified two shared gene ontologies, an ion transport regulation and a positive ion transport regulation. Within these ontologies, two genes were further identified as being involved with cholinergic neuronal signal transmission, *GABBR1* and *KCNN2*. The role of ion transport in neurological signaling is complex, as it features a series of ion transport enablers, which boost ion transport and depolarize membranes, as well as counter-transporters, which work to maintain membrane hyperpolarization. These ion transport players in cholinergic and GABA-ergic pathways amplify or inhibit transmission of synaptic signals, respectively. As a result of these interactions, a carefully tuned balance emerges. Potential implications of the presence of *GABBR1* and *KCNN2* variants in the context of autism and sensory processing are discussed below. Further consideration is given to how operation of these pathways may be altered during acetylcholine deficiency resulting from an inadequate supply of dietary choline.

### *GABBR1*/GABA_B__1_ Implications

The *GABBR1* gene encodes for the membrane protein known as GABA_B__1_ (or sometimes GABA_B_). This membrane protein, which constitutes the metabotropic class of GABA-binding membrane-bound receptors, functions at a slow, steady level, in partnership with another membrane-bound GABA-binding protein, GABA_B__2_. Through activation of potassium ion channels that release potassium (K^+^) out of the neuron and inhibition of calcium ion (Ca^2+^) channels which permit passage of Ca^2+^ ions into the neuron, this set of proteins brings about membrane hyperpolarization. In this circuit, GABA–GABA_B__1_ binding activity serves an inhibitory function, which counters the possibility of excitation of membrane depolarized neurons. This functionality helps to modulate a magnitude of incoming stimulation by inhibiting sensory signals transmitted by cholinergic membrane depolarization. Thus, activation of neuronal action potentials and a subsequent, consistent state of neuronal overexcitation are prevented (Figure 5).

Additionally, GABA_B__1_ receptor binding initiates a cascade which eventually results in the inhibition of acetylcholinesterase—the enzyme that degrades acetylcholine in the synapse. This indicates a possibility that GABA_B__1_ receptor binding activity may at least in part regulate the size of the acetylcholine pool available for cholinergic signaling.

The *GABBR1* variant associated with autism—variant rs740883—is an intronic variant in which a thymine replaces an adenine (Liu et al., 2020). This single-nucleotide polymorphism (SNP) occurs in approximately 9–10% of individual human genomes, and is located within a 3 prime untranslated region, commonly associated with a stability of a transcript. *GABBR1*-encoded GABA_B__1_ receptors are expressed at lower levels in autistic brains, in both the superior frontal and parietal cortices, and in the cerebellum (Fatemi et al., 2009, 2010). Levels of related GABA-ergic biomarkers also differ in the brains of people with an autism diagnosis, when compared to levels of GABA-ergic biomarkers in control brains (Blatt and Fatemi, 2011). While the autism-associated *GABBR1* intron variant discussed here is discovered too recently to be analyzed for its relation to these epigenetic differences, examining the possibility that this variant may explain the observed autism-specific differences in GABA-ergic transcriptomic regulation described in the scientific literature is warranted.

### Impact of GABA/GABA_B__1_ Binding on the Synaptic Supply of Acetylcholine

GABA is a common neuromediator which often functions in tandem with acetylcholine, in either inhibitory or excitatory roles that span learning, memory, sensory signal transmission, neuromuscular function, and cardiac function. In the hippocampus/somatosensory cortex, the mammalian central nervous system, and elsewhere in the body, GABA and acetylcholine may be co-released, in a relationship which is not yet fully understood (Bianchi et al., 1982; Kusunoki et al., 1984; Granger et al., 2016; Takács et al., 2018). Both of these mediators rely upon vesicle-based exocytosis to be released into the synapse, with GABA and acetylcholine transporting vesicles within the same presynaptic vesicle pools being filled independently of each other (Takács et al., 2018).

Notably, the binding of GABA_B__1_ to its ligand impacts available interneuronal acetylcholine supply (Figures 5, 6A). More specifically, GABA–GABA_B__1_ binding inhibits a cascade in which the transcription factor CREB1 boosts expression of acetylcholinesterase (ACHE), an enzyme that degrades acetylcholine in the interneuronal junction, thereby lessening the magnitude of cholinergic signal. If GABA–GABA_B__1_ binding activity were to *decrease* as a result of a presence of a genetic variant in a *GABBR1* gene—for example, rs740883, which is strongly associated with autism—an increase in acetylcholinesterase production would ensue after being prompted by an increase in the binding of CREB1 *ACHE* promoter. In this scenario, the increased supply of acetylcholinesterase would degrade acetylcholine in the interneuronal junction faster, thus suppressing cholinergic signaling to a greater extent than in the brain with wildtype *GABBR1*. Figure 6A systematically depicts hypothesized impact of *GABBR1*’s autism-associated intronic variant on GABA-ergic and cholinergic signaling.

### Impact of Acetylcholine Deficiency on Cholinergic Output, and Hypothesized Interplay With *GABBR1* Genetics

Both our modeling and limited experimental evidence suggest that insufficient dietary intake of a choline may contribute to a reduced acetylcholine supply in the brain (Cohen and Wurtman, 1976). The combination of a dietary choline deficiency with a *GABBR1* variant may result in cholinergic signaling being lowered further, or even markedly impaired. Moreover, given that GABA sometimes co-transmits with acetylcholine, reduced acetylcholinergic signaling may also be tied to the temporal restriction on the GABA-driven neuronal inhibition as a secondary event. Sensory processing depends upon function of both of these neurotransmitters, so it is reasonable to extrapolate that the combination of dysregulated ion transport due to the presence of *GABBR1*/GABA_B__1_ variants and dietary-driven acetylcholine deficiency may result in altered sensory processing for the individual in question.

### *KCNN2*/SK2 Channel Implications

The gene *KCNN2* encodes for the membrane-bound SK2 calcium-activated potassium ion channel, which modulates the excitability of neurons and neuromuscular activity. After being activated through co-located acetylcholine nicotinic receptors, SK2 channel modulates cholinergic signals through neuronal membrane repolarization (Figure 7A). SK2 channels are abundant throughout the body, including the brain and the cardiorespiratory system (Gu et al., 2018). The SK2 channel encoded by *KCNN2* is associated with sensory processing in a variety of contexts, with much research focusing on auditory processing with respect to cholinergic nicotinic receptors in the cochlear hair (Elgoyhen et al., 2001, 2009; Kong et al., 2008; Murthy et al., 2009),vestibular awareness (Wangemann, 2002), and nociception (Bahia et al., 2005; Mongan et al., 2005; Pagadala et al., 2013; Hipólito et al., 2015; Thompson et al., 2015).

### Impact of Acetylcholine Supply on the Function of *KCNN2*/SK2 Channel

As discussed above in relation to the GABA_B__1_ receptor, dietary choline deficiency may markedly reduce the pool of acetylcholine in the brain (Cohen and Wurtman, 1976). It follows that diminished binding of the acetylcholine to cholinergic receptors may reduce activation of co-located SK2 channels. In turn, reduced acetylcholine supply in the brain may require increased sensory input, which, in practical terms, translates into sensory hyposensitivity, or sensory underresponsiveness. Reduced signaling may alter the closely-tied counterfeedback of SK2 ion transport signal modulation.

### Impact of Acetylcholine Deficiency on Cholinergic Output, and Its Possible Interplay With *KCNN2* Genetics

Similar to the autism-associated epigenetic regulation of *GABBR1* observed in the literature, an autism-specific epigenetic regulation of SK2 ion transport activity was noted. In the brains of people with an autism diagnosis, cholinergic nicotinic receptors are expressed at lower levels when compared to that in neurotypical brains (Lee et al., 2002; Andersen et al., 2013). A lower level of available cholinergic receptors may translate to fewer opportunities for acetylcholine binding, and, as collateral, the normally colocalized SK2 channels may also fire less frequently, cumulatively altering the degree of membrane repolarization, and therefore, the timing of subsequent cholinergic sensory signaling.

Because SK2 signaling depends upon the signals from co-located cholinergic nicotinic receptors, diminished or altered SK2 activity arising from the presence of autism-associated *KCNN2* coding variant(s) may be compounded by the synaptic acetylcholine deficiency. Thus, dietary choline deficiency may ultimately exacerbate genetic weakness in the SK2 activity, resulting in altered sensory processing, possibly resulting in sensory over-responsiveness, under-responsiveness, or a combination of both, for a given autistic person.

### rs13188074 Variant May Influence *KCNN2* Splicing Pattern

Currently, there is a little evidence of direct impact of intronic variants in *GABBR1* and *KCNN2* on the function of the respective proteins. ASSP analysis hints that, in case of *KCNN2*, the change in a strength of a splice site may influence an amount of functional mRNA encoding for full-size *KCNN2*. Experimental gauging of the impact of *KCNN2* variant upon sensory processing is warranted.

### Targeting Cholinergic and GABA-ergic Signaling Pathways May Modulate Sensory Processing in Autism

The models indicated in Figures 2, 3 are consistent with extant literature examining the possibility of cholinergic pharmacotherapy of autism, as increasing the pool of available interneuronal acetylcholine may improve function in several domains related to cholinergic signaling activity and related ion transport dynamics. For example, acetylcholinesterase inhibitors galantamine and memantine, which are typically prescribed for Alzheimer’s disease patients, are also actively explored in autism (Maire and Wurtman, 1984; Hertzman, 2003; Nicolson et al., 2006; Ghaleiha et al., 2014; Rossignol and Frye, 2014; Rahman et al., 2018). A systematic review examining the impact of these drugs in individuals with autism identified an improvement across several domains, including communication and social interaction, and a decrease in disruptive behavior, hyperactivity, inattention, and irritability (Rossignol and Frye, 2014). Many of these domains are linked to sensory processing difficulties (Ashburner et al., 2008; Sanz-Cervera et al., 2015). In the present study, we posit that variant function of *GABBR1* may lead to a smaller pool of available acetylcholine (Figure 5). For patients with *GABBR1* variants, including individuals with autism, prescription of acetylcholinesterase inhibitors such as galantamine or memantine may counter the disturbance in GABA/*GABBR1*/acetylcholinesterase cascade, by boosting the synaptic acetylcholine levels to match these seen in neurotypical brains.

In the context of addiction, the literature supports a relationship between acetylcholinergic transmission and genetic variation of *GABBR1*. Individuals with genetics variants of the *GABBR1* locus are more likely to develop dependency upon a major cholinergic nicotinic receptor agonist, nicotine. This observation highlights the complexity of the influence of genetic *GABBR1* variation upon acetylcholinergic function (Li et al., 2009; Li, 2018). Notably, nicotinic receptors have been of particular interest as a therapeutic target in autism, as their stimulation affects both working memory and executive functions. In a placebo-controlled trial, nicotine patches have been investigated as a mean to improve sleep and to address aggressive behavior in autistic adults (Deutsch et al., 2015; Olincy et al., 2016; Lewis et al., 2018; Deutsch and Burket, 2020).

The evidence for pharmacological intervention on GABA_B__1_ upon autism-related sensory domains is equivocal. GABA_B__1_ receptor agonists such as arbaclofen have been investigated through evaluation of their efficiency across measurable features of irritability, lethargy, and social responsiveness (Veenstra-VanderWeele et al., 2017). When examining a specific sensory subdomain—auditory processing—a subset of teenagers with autism taking arboclofen were found to exhibit improved magnetoencephalographic (MEG) measures of their auditory response (Roberts et al., 2019).

It is of certain importance that in addition to pharmacological means, cholinergic transmission may also be influenced by change in the dietary intake of the choline, one of the common nutrients abundant in milk, liver, eggs, and peanuts. As discussed, many Americans do not consume the daily AI level of choline, and indeed, recent surveys indicate that a majority of a sample of autistic children (60–93%) do not meet their choline AI level recommended respective of age (Hamlin et al., 2013; Wallace and Fulgoni, 2016). A number of autism-associated traits such as preference for routine, aversion to particular foods, as well as sensory aversions to particular smells, textures, or temperatures are known to profoundly shape the diets of people on the autism spectrum (Dunn, 1997; Baranek et al., 2006; Cermak et al., 2010; Nadon et al., 2011; Kral et al., 2013; Hubbard et al., 2014; Sanz-Cervera et al., 2015; Bogdashina and Casanova, 2016; Boterberg and Warreyn, 2016; Crasta et al., 2016; Bitsika and Sharpley, 2018; Weeden, 2019). Because of that, the availability of an adequate pool of acetylcholine for cholinergic signaling may be a legitimate concern, particularly in light of the potentially exacerbated need for proper function of sensory signaling pathways discussed above.

### Dietary Choline: A Low-Risk Intervention to Support Acetylcholine Supply, Cholinergic Signaling, and Sensory Processing in Autism

The recommended dietary consumption levels for choline are set by the Food and Nutrition Board of the National Academies as assessed by measuring serum alanine aminotransferase levels reflecting overall liver function. Whether or not the choline at AI level would be sufficient to modulate sensory processing symptoms is not known. It is notable, though, that most Americans do not achieve even this level of the choline intake (Wallace and Fulgoni, 2016). For autistic individuals, who experience particular difficulty in meeting dietary guidelines for choline through diet alone, choline supplementation may provide an alternate effective means of ameliorating daily choline intake levels. As opposed to pharmacological interventions, using the dietary modifications or choline supplements is considered low risk, because neither dietary nor supplementary forms of choline are known to interact with medications. Additionally, overconsuming choline through diet or supplementation to the point of excess—which is defined by the tolerable upper limit of 3,500 mg/day—would require Americans to consume more than seven times their recently estimated mean intake levels of choline, which is currently at less than 500 mg/day for all age groups (Institute of Medicine (US) Standing Committee on the Scientific Evaluation of Dietary Reference Intakes and its Panel on Folate, Other B Vitamins, and Choline, 1988; Wallace and Fulgoni, 2016; Wallace et al., 2018).

To call dietary intervention for choline status “low risk” does not negate the challenges specific to supporting the increased consumption of choline in autistic children and adults. The research and development which is required to determine targeted, effective ways for elevating choline consumption and identifying personalized intake baselines sufficient for fulfilling physiological need for choline in this population is considerable. As with any autism research, investigations should purposefully include representatives and researchers with an autism diagnosis who can help to shape such interventions optimally for autistic children and adults (Chown et al., 2017; Hoekstra et al., 2018; Lebenhagen, 2019; Cascio et al., 2020; Hogan et al., 2020).

### Future Research Directions

The autism-associated *GABBR1* and *KCNN2* genetic variants have been highlighted as such only recently, and therefore there is an opportunity to collect more definitive experimental data, which can inform the modeling of sensory processing dynamics as they relate to these variants. Studies of *GABBR1* and *KCNN2* in autism should examine possibilities such as structure/function differences in the autism-associated variant version of these membrane proteins, as well as measurement of *GABBR1* and *KCNN2* expression levels in those presenting with the respective autism-associated variants.

Because of the relative likelihood that autism-associated intron variants such as those found in *GABBR1* and *KCNN2* would result in transcriptomic changes, transcriptomics should be a primary focus of future research on these genetic variants’ impact. Because of the measured difference in GABA_B__1_ protein expression in autism, examining a potential link between the autism-associated *GABBR1* variant and these expression differences should be a priority. Similarly, SK2 channel expression levels in autism should be examined with respect to this SK2 channel variant. Any transcriptomic differences which would be found to be associated with these genetic variants would set the foundation for more refined examination of impact upon cholinergic signal transmission and modulation.

In terms of further examination of potential alterations to autism-related variant versions of GABA_B__1_ and SK2 channel structure and/or function, there are several tools and methods available, which vary in scope and performance. For example, if it can be determined that a reading frame shift has occurred as a consequence of an altered splice site, basic computational homology modeling may be helpful. Without the context of a frame shift, if structural differences are still suspected for the variant version of either of these proteins, validated structural assessment through means of electron microscopy, NMR spectroscopy, and X-ray crystallography remains as resource-intensive possibilities. Additionally, new structure prediction AI presented by AlphaFold, exhibiting unprecedented accuracy in structural prediction, could be considered as a potential future tool (DeepMind, 2020). Additionally, if structural differences were uncovered, molecular dynamics modeling may provide insights into the relative binding frequencies and strength in wildtype vs. variant versions of GABA_B__1_ receptors and SK2 channels. If these models provide actionable information, they may merit further development in terms of broadening molecular dynamics model scope to include various elements from the cholinergic sensory signal pathways and cascades discussed in this study. Computational modeling and pathway exploration may continue to provide support and context as researchers continue to explore potential impact of these genetic variants upon sensory processing in autism.

In terms of clinical interventions, while available evidence on dietary interventions for autism remains limited, this work provides mechanistic support for further exploration of exogenous choline—through diet or supplementation—as a potential low-risk sensory processing support intervention. Future clinical interventions in humans will *de facto* require the input of multidisciplinary teams of researchers and stakeholders—including those with autism diagnoses—to effectively develop and test autism-tailored clinical interventions that carefully measure inputs through diligent nutritional assessment, and outcomes such as standardized sensory processing scores, and choline activity measured through functional magnetic resonance imaging (fMRI). Such teams may include researchers from a broad range of fields including nutrition, neuroscience, occupational therapy, bioinformatics, and computational biology. Additionally, choline-related biomarkers such as serum alanine aminotransferase may provide useful outcome measurements, particularly in cases where nutritional assessment indicates risk of deficiency.

## Conclusion

In connecting dietary choline intake as a mediator of acetylcholinergic pathways, especially for sensory signal ion transport in the context of two autism-associated genes, GABBR1 and KCNN2, this study is consistent with the growing evidence base concerning the role of choline in pre- and postnatal nutrition and neurodevelopment. In our opinion, clinical evaluation of choline intake interventions with respect to validated sensory processing scores is warranted in future studies whose age ranges include children, teens, and adults.

Because of the physically, mentally, and logistically demanding requirements involved in collection of experimental data through clinical trials, particularly from participant individuals with autism and their families, it is incumbent upon researchers to ensure that clinical trials examining dietary status, genetic variants, and sensory processing are effective, efficient, and respectful of their participants. Advance planning and implementation for these clinical trials is therefore at a premium. Accordingly, in preparation for clinical trials, research employing computational biology, bioinformatics, and predictive modeling should also be considered as essential precursor elements in examining the intersection of dietary choline status with sensory processing in the context of autism-associated genes.

## Data Availability Statement

The autism GWAS dataset is available as a supplementary file for the original article (Wu et al., 2020).

## Author Contributions

AO, AB, and MS were responsible for the conception of the project and editing the final manuscript. AO carried out analyses and interpretation with input from all other authors and drafted the manuscript with feedback from all other authors. All authors contributed to the design.

## Conflict of Interest

The authors declare that the research was conducted in the absence of any commercial or financial relationships that could be construed as a potential conflict of interest.

## Footnotes

1 www.pathwaystudio.com
